# Genomic and Non-genomic Action of Neurosteroids in the Peripheral Nervous System

**DOI:** 10.3389/fnins.2020.00796

**Published:** 2020-07-29

**Authors:** Alessandra Colciago, Veronica Bonalume, Valentina Melfi, Valerio Magnaghi

**Affiliations:** Department of Pharmacological and Biomolecular Sciences, Università degli Studi di Milano, Milan, Italy

**Keywords:** neuroactive steroid, allopregnanolone, GABA, myelin, Schwann cell, dorsal root ganglia

## Abstract

Since the former evidence of biologic actions of neurosteroids in the central nervous system, also the peripheral nervous system (PNS) was reported as a structure affected by these substances. Indeed, neurosteroids are synthesized and active in the PNS, exerting many important actions on the different cell types of this system. PNS is a target for neurosteroids, in their native form or as metabolites. In particular, old and recent evidence indicates that the progesterone metabolite allopregnanolone possesses important functions in the PNS, thus contributing to its physiologic processes. In this review, we will survey the more recent findings on the genomic and non-genomic actions of neurosteroids in nerves, ganglia, and cells forming the PNS, focusing on the mechanisms regulating the peripheral neuron-glial crosstalk. Then, we will refer to the physiopathological significance of the neurosteroid signaling disturbances in the PNS, in to identify new molecular targets for promising pharmacotherapeutic approaches.

## Introduction

The importance of endogenous neurosteroids for the control of the peripheral nervous system (PNS) become increasingly relevant in the last decades. Since the 1980 last century, when Baulieu and colleagues (Baulieu, 1997) introduced the term “neurosteroids” to indicate steroids that were synthesized *de novo* in the brain, also the PNS has been referred as an important target of their action. In the PNS, the neuroactive steroids (comprising the aforementioned neurosteroids as well as the hormonal steroids) are synthesized and/or metabolized in active forms and exert important physiopathologic functions.

**FIGURE 1:**
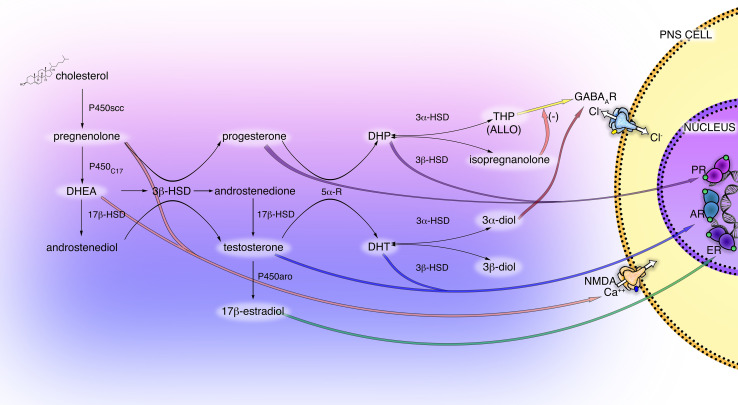
Scheme of principal neuroactive steroid biosynthetic and metabolic pathways, and their receptor interactions in the PNS. The main steroidogenic enzymes, metabolites, and receptors modulating neuroactive steroid action are present in the cells of the PNS (i.e., Schwann cells and DRG neurons). These steroidogenic pathways, through the intermediate pregnenolone (PREG), dehydroepiandrosterone (DHEA) and androstenediol lead to the synthesis of progestogens (PROG, progesterone; DHP, dehydroprogesterone; THP, tetrahydroprogesterone also called ALLO, allopregnanolone; isopregnanolone), androgens (T, testosterone; DHT, dihydrotestosterone; 3α-diol, 5α-androstane-3α,17β-diol; 3β-diol, 5α-androstane-3α,17β-diol) and estrogens (E2, 17β-estradiol). P450 side-chain cleavage enzyme (P450scc); 17α-hydroxylase/17,20-lyase (P450C17); 17β-hydroxysteroid-dehydrogenase (17β-HSD); 5α-R, 5α-reductase; 3α-hydroxysteroid-dehydrogenase (3α-HSD); 3β-hydroxysteroid-dehydrogenase (3β-HSD); P450 aromatase (P450aro). These neuroactive steroids may act through classical receptor (PR, progesterone receptor; AR, androgen receptor; ER estrogen receptor) or non-classical receptor (GABA type A receptor; NMDA receptor), see text for details.

The neurosteroidogenic machinery includes a set of enzymes (Figure 1), starting from P450 side-chain cleavage (P450scc), which convert cholesterol into pregnenolone (PREG) in mitochondria, moving to the 3β-hydroxysteroid-dehydrogenase (3β-HSD), that converts PREG into progesterone (PROG) or the 17α-hydroxylase/17,20-lyase (P450C17), converting PREG into dehydroepiandrosterone (DHEA). These steroids may be then metabolized into androgens, androstenediol, androstenedione and testosterone (T). Furthermore, by the action of the enzyme P450 aromatase the androgens androstenedione and T are converted into the estrogens estrone and 17β-estradiol (E2), respectively.

Most of these enzymes, as well as their metabolic products PROG, PREG, T, DHEA, and E2, have been found in PNS (Caruso et al., 2008). In particular, the glial Schwann cells (SC) of the PNS possess the enzymatic machinery required to produce the neurosteroids: P450scc, 3β-HSD, etc., (Celotti et al., 1992; Melcangi et al., 2001; Schumacher et al., 2001). The activity of some steroidogenic enzymes in SC is neuronal dependent, such as the 3β-HSD, that raised in the presence of neurons (Chan et al., 2000; Robert et al., 2001). Dorsal root ganglia (DRG) express the P450scc and 3β-HSD but not other steroidogenic enzymes, indicating that other autocrine or paracrine mechanisms might influence the steroidogenesis in the soma of primary sensory neurons (Schaeffer et al., 2010). Additionally, the enzymatic complexes formed by the 5α-reductase (5α-R) and the 3α-hydroxysteroid-dehydrogenase (3α-HSD) was found in the PNS (Figure 1), primarily in SC (Melcangi et al., 1990; Celotti et al., 1992; Melcangi et al., 1992, 1999b). This enzymatic complex converts steroids possessing the delta(4)-3-keto configuration into their more active 5α-3α-reduced metabolites, the so-called neuroactive steroids (Celotti et al., 1992). Thereby, the PROG is converted into dihydroprogesterone (DHP) and then into 5α-pregnan-3α-ol-20-one, also named tetrahydroprogesterone (THP) or allopregnanolone (ALLO); similarly, the steroid T is converted into dihydrotestosterone (DHT) then into 5α-androstane-3α,17β-diol (3α-diol). The 5α-reduced intermediate metabolite DHP and DHT can be further converted, by the enzyme 3β-HSD, into 5α-pregnan-3β-ol-20-one (also named isopregnanolone) or 5α-androstane-3β,17β-diol (3β-diol), respectively (Giatti et al., 2015; Figure 1).

The PNS showed also the presence of other important factors supporting *de novo* local synthesis of neuroactive steroids. For instance, those factors regulating the translocation of cholesterol into the mitochondria, likely the steroidogenic acute regulatory protein (StAR) and the translocator protein of 18 kDa (TSPO), as well as the liver X receptor (LXR), were found to be present and active in peripheral nerves. TSPO was formerly considered as a crucial protein for steroid biosynthesis (Li and Papadopoulos, 1998). More recent and debated observations, however, argued the significance of TSPO in steroidogenesis and evidenced its involvement in pathological conditions, like inflammation, apoptosis and neurological diseases (e.g., Alzheimer’s disease or multiple sclerosis); indeed, TSPO seems to be expressed only in response to insults and pathological states (Bonsack and Sukumari-Ramesh, 2018). In the PNS, the activation of TSPO with the specific ligand Ro5-4864 improved the levels of neuroactive steroids and exerted neuroprotective effects in the peripheral nerves of streptozotocin (STZ)-induced diabetic rats (Giatti et al., 2009). LXR is a ligand activated transcription factor belonging to the nuclear receptor superfamily. It is important for cholesterol biosynthesis, serving as a sensor that prevents the excessive intracellular accumulation of cholesterol (Jakobsson et al., 2012). In the PNS, its activation by specific ligands induces neuroactive steroid synthesis in the sciatic nerve of STZ-induced diabetic rats, thus ameliorating diabetes-induced neuropathy (Cermenati et al., 2010).

Interestingly, the synthesis and the levels of neuroactive steroids proved sexually dimorphic, in physiologic states (Melcangi et al., 2016), as well as in peripheral neurodegenerative conditions, such as the diabetic neuropathy (Pesaresi et al., 2010). For instance, in the sciatic nerve of STZ-rats the levels of PREG, T, DHT, and 3α-diol decreased in males, whereas the levels of PROG, THP and isopregnanolone drop down only in female (Pesaresi et al., 2010). In the same rat model of diabetic neuropathy, the gonadectomy ameliorates the nerve alterations in females but not in males (Pesaresi et al., 2011a).

In this review, we will survey the recent findings of the classical and non-classical, genomic and non-genomic action of neuroactive steroids in peripheral nerves, ganglia and cells forming the PNS, thereby focusing on the mechanisms regulating the peripheral neuron-glial crosstalk.

## Mechanisms of Action of Neuroactive Steroids

In the PNS, the neuroactive steroids exert several biologic functions, modulating the mitogenic activity, cell proliferation, myelination process, nerve repair, and axonal conduction.

The neuroactive steroid actions occur through either “classical” or “non-classical” receptors, which localized both in the neuronal and in the glial compartment (i.e., SC) of the PNS. The classical action is generally genomic and consists of the binding to intracellular receptors in the target cells, followed by the regulation of gene transcription (Slater et al., 1994). Conversely, the non-classic action is more rapid and involves the modulation of membrane receptors, such as neurotransmitter and neurotrophin receptors, ion channels or the newest membrane steroid receptors (Brann et al., 1995; Barabas et al., 2018). Commonly, among the neurotransmitter receptors affected by neuroactive steroids, there are the γ-aminobutyric acid (GABA) and the N-Methyl-D-aspartate (NMDA) receptors (Lambert et al., 1996; Rupprecht et al., 2001; Monnet and Maurice, 2006; Sedlacek et al., 2008). Moreover, the family of steroid membrane receptors includes specific receptors for estrogens, androgens, glucocorticoids and progestogens (Levin, 2011). Whether these receptors are the classical nuclear receptor, which localizes on the cell membrane, or distinct receptors characterized by different proteins is still a matter of debate.

Interestingly, the capability of neuroactive steroids to interact with classical rather than non-classical receptors is ancillary to their conversion into active compounds. For instance (Figure 1), the progestogens PROG and DHP mainly exert classical activity through the genomic PROG receptor (PR), while their metabolite ALLO fulfills a non-classical activity via the GABA type A (GABA-A) receptor. Indeed, ALLO is one of the most re-known and important GABA-A receptor modulators (Lambert et al., 2009; Faroni and Magnaghi, 2011), while the progestogen metabolite isopregnanolone has proved to antagonize the effect of ALLO at GABA-A receptor (Wang et al., 2002). Similarly, androgen metabolites exert classical and non-classical actions. For instance, (Figure 1) 3α-diol activates the GABA-A receptor, whereas 3β-diol is an agonist of the estrogen receptor (ER) beta (ERβ) (Lambert et al., 2003; Handa et al., 2008).

Evidence on the involvement of all these receptors in the different physiopathologic states affecting the PNS has been fully reported.

## Genomic Actions of Neuroactive Steroids in PNS

Classical intracellular steroid receptors, such as PR, ER, androgen receptor (AR), glucocorticoid receptor (GR), and mineralocorticoid receptor (MR) were found in peripheral nerves (Magnaghi et al., 1999, 2001; Jordan et al., 2002; Shaqura et al., 2016) as well as in SC (Neuberger et al., 1994; Jung-Testas et al., 1996; Groyer et al., 2006; Faroni and Magnaghi, 2011; Shaqura et al., 2016) and DRG (Luo et al., 2008; Dong et al., 2012; Shaqura et al., 2016; Figure 1). Generally, these classic receptors bind, respectively, the progestogens PROG and DHP, the estrogens E2 and estrone, the androgens DHEA, T and DHT, the gluco/mineralocorticoids corticosterone, dehydrocorticosterone, and deoxycorticosterone (Slater et al., 1994; Prough et al., 2016).

In the PNS, E2 promoted the proliferation and differentiation of SC, *in vitro* and *in vivo*, thus fostering the myelination process (Chen et al., 2016; Gu et al., 2018). By the way, some of these effects were inhibited by the antagonist of the genomic ER type α (ERα) and type β (ERβ) receptors, ICI182780 and 2-phenyl-3-(4-hydroxyphenyl)-5,7-bis(trifluoromethyl)-pyrazolo[1,5-a]pyrimidine (PHTPP), respectively, highlighting a classical genomic mechanism. Nevertheless, the specific block of the intracellular signaling cascade of the extracellular signal-regulated protein kinase 1/2 (ERK1/2) or AKT (commonly downstream the activation of the membrane receptor) evidenced that also these pathways may occur, and suggested that the mechanisms are complicated, likely involving both genomic and non-genomic actions (Gu et al., 2018). Besides, also PROG was able to promote SC proliferation, and its effects appeared sex-specific (Jung-Testas et al., 1993). E2 was effective in males, while PROG promotes SC proliferation only in females. These actions implied genomic mechanisms since they were blocked by specific ERα and PR antagonists, ICI128780 and zk112994, respectively, (Fex Svenningsen and Kanje, 1999).

In the PNS, the effect of neuroactive steroids been extensively studied on the myelination and re-myelination processes has been extensively studied. The glucocorticoid corticosterone stimulated the expression of the two most important proteins of the peripheral myelin (Desarnaud et al., 2000): the glycoprotein P0 (P0) and the peripheral myelin protein of 22 kDa (PMP22). However, progestogens were proved as the more compelling steroids able to regulate the PNS myelination. PROG, DHP, and ALLO stimulated the expression of P0 and PMP22 in the sciatic nerve of young and old male rats (Melcangi et al., 1998, 1999a, 2000b; Faroni and Magnaghi, 2011). In *in vivo* models, PROG, DHP and ALLO proved able to reduce the age-associated myelin abnormalities in the sciatic nerve of elderly rats (Azcoitia et al., 2003) and stimulated the re-myelination of injured nerves, in a model of nerve cryolesion or transection (Koenig et al., 1995; Melcangi et al., 2000a). Furthermore, in a model of guided facial nerve regeneration, PROG increased the SC proliferation, myelination as well as the number of nerve fibers (Chavez-Delgado et al., 2005). The effects of PROG and DHP on P0 and PMP22 levels occurred also in SC cultures (Desarnaud et al., 1998; Melcangi et al., 1998, 1999a; Magnaghi et al., 2001), indicating a direct classical genomic effect of progestogens on these cells. In accordance, we proposed that the complicated and long-term effects of PROG, DHP and ALLO (after its retro-conversion into DHP, within PNS cells; see Figure 1) in modulating the expression of protein P0 are linked to the interaction with the PR expressed in SC (Magnaghi et al., 2001). The specific PR antagonist mifepristone (RU38486), indeed, blocked the effects of PROG, DHP and ALLO on the P0 levels (Melcangi et al., 2003), corroborating the genomic mechanism. Nevertheless, the rapid effect of ALLO on PMP22 levels seemed due to an interaction with the GABA-A receptor expressed in SC (see the chapter below).

The classical genomic effect on P0 was sustained by the presence of putative PROG responsive elements on the P0 gene (Magnaghi et al., 1999) and by the involvement of the steroid nuclear receptor coactivator SRC1 in the regulation of P0 expression (Cavarretta et al., 2004). Interestingly, the genomic action exerted by progestogens on the PNS myelin proteins, that is P0, PMP22 and myelin associated glycoprotein (MAG), was sex-specific; in fact male rats resulted more responsive to the genomic effects of PROG and DHP (Magnaghi et al., 2006b). It was highlighted that PROG coordinates also the initiation of the PNS myelination, because it increases the expression of some basic transcription factors priming the SC myelination, such as early growth response 2 EGR2 (EGR2/Krox-20), early growth response (EGR1/Krox-24), early growth response 3 (Egr-3), SRY-box10 (Sox10) and Fos B (Guennoun et al., 2001; Mercier et al., 2001; Magnaghi et al., 2007). At least in the case of Krox-20, the presence of putative PROG responsive elements in the gene promoter support a PR-mediated genomic effect (Magnaghi et al., 2007).

Other findings suggested that the glycoprotein P0 is also under the control of classical AR. Gonadectomy of adult male rats induced a decrease in myelin protein P0, whereas DHT enhanced the P0 levels in sciatic nerve of normal animals (Magnaghi et al., 1999). This effect attested the capacity of androgens to participate in the control of peripheral myelination, however, since the SC do not express AR (Magnaghi et al., 1999), the effect was supposed to be indirectly mediated via the neuronal compartment. The androgens efficacy may be ascribed to the crosstalk between SC and the axon, hypothesizing the transfer of vesicles likely containing the receptors (Grossfeld et al., 1988; Lopez-Verrilli and Court, 2012), or the involvement of motoneurons which express the AR (Jordan et al., 2002). The finding that genomic effects of neuroactive steroids on SC are indirectly mediated by the neuronal compartment is supported by the observation that the PR antagonist mifepristone induced an axonal impairment during the development, determining a significant reduction of axon diameter (Melcangi et al., 2003). In accordance, the PROG enhancement of myelin formation was shown in an *in vitro* co-culture model of SC-DRG neurons, corroborating the requirement of the neuronal compartment for the progestogen action (Chan et al., 2000).

Neuroactive steroids, mainly PROG and DHP, also exert neuroprotective and pro-regenerative effects in case of neurodegenerative pathologies of the PNS, such as nerve traumatic injuries (i.e., cryolesion, transection or crush) or diabetic neuropathy (Koenig et al., 1995; Melcangi et al., 2000a; Chavez-Delgado et al., 2005; Leonelli et al., 2007; Roglio et al., 2008). For instance, PROG and DHP, likely through genomic mechanisms involving the PR, are able to counteract the decrease of P0 and PMP22 expression induced in the STZ model of diabetic neuropathy (Leonelli et al., 2007). Both neuroactive steroids decreased the number of altered fibers (i.e., presenting myelin infoldings) in the sciatic nerve of STZ neuropathic rats (Veiga et al., 2006), still corroborating the neuroprotective role of PR. In parallel, also the androgen DHT proved able to increase the P0 mRNA levels in the sciatic nerve of STZ neuropathic rats, likely via AR-mediated mechanisms (Roglio et al., 2007). In the same model of STZ-induced neuropathy, DHP and DHT improved another hallmark of diabetic neuropathy, promoting the changes in Na^+^-K^+^ ATPase activity (Leonelli et al., 2007; Roglio et al., 2007). In diabetic rats, also the treatment with DHEA exerted neuroprotective effects, mostly in females rather than in male animals (Pesaresi et al., 2011b). DHEA was effective following rat sciatic nerve transection, whereas it reduced the extent of denervation atrophy stimulating the earlier onset of axonal regeneration (Ayhan et al., 2003). Following traumatic nerve crush injury, DHEA and E2 promote the fast recovery of gait along with an enhancement of myelinated fibers (Gudemez et al., 2002; Islamov et al., 2002). Moreover, also T was capable to accelerate the functional recovery following rat sciatic nerve crush (Kujawa et al., 1993; Brown et al., 1999).

Evidence of GR-dependent induction of gene transcription was found in adult DRG neurite, which grew in response to stress or glucocorticoid treatment. This phenomenon exacerbates the effect of acute systemic stress on neuronal plasticity and PNS regeneration (Lerch et al., 2017). Importantly, a putative role of GR in regulating peripheral nociception has been proposed. This hypothesis was corroborated by the GR localization, which was found predominantly in peripheral nociceptive unmyelinated C-fiber and Aδ lightly myelinated fibers (Shaqura et al., 2016).

## Non-Genomic Action of Neuroactive Steroids in PNS: Role of Membrane Steroid Receptors

The steroid membrane receptors mediate the rapid (second to minutes) non-classical, non-genomic action of neuroactive steroids, occurring at the cell surface of neurons and glial cells. To date, it consists of specific receptors for estrogens (membrane ER, mER), androgens (membrane AR, mAR) glucocorticoids (membrane GR, mGR) and progestogens (membrane PR, mPR). These receptors mostly belong to the G protein-coupled receptor (GPCR) family and activate a plethora of intracellular signaling cascade (Levin, 2011). Recent studies investigated the presence of some membrane receptors in the PNS, focusing primarily on the subfamilies mER and mPR.

The GPR30, named GPCR ER-1 (GPER1), is a non-nuclear ER located on the cell membrane, which binds E2 with high affinity and potency, thus mediating non-genomic events (Thomas et al., 2005). DRG, autonomic pelvic ganglia and sensory trigeminal ganglia express GPR30, which modulation by the specific G1 agonist induced a membrane depolarization (Dun et al., 2009). In the PNS, however, some rapid estrogenic effect seemed to be due to the non-genomic action of classic ERα, likely translocated to the cell membrane. For instance, mouse DRG neurons express membrane associated ERα, producing a rapid attenuation of ATP-induced Ca^++^ signaling, likely a mechanism involved in gender-specific pain perception (Chaban and Micevych, 2005). Another study underlined the cytoprotective potential of E2 on the transplanted SC in a model of spinal cord injury (Siriphorn et al., 2010). Protection was not inhibited by classical ER antagonist ICI 182780, suggesting that non-genomic mechanisms involving mER may occur (Siriphorn et al., 2010).

**FIGURE 2:**
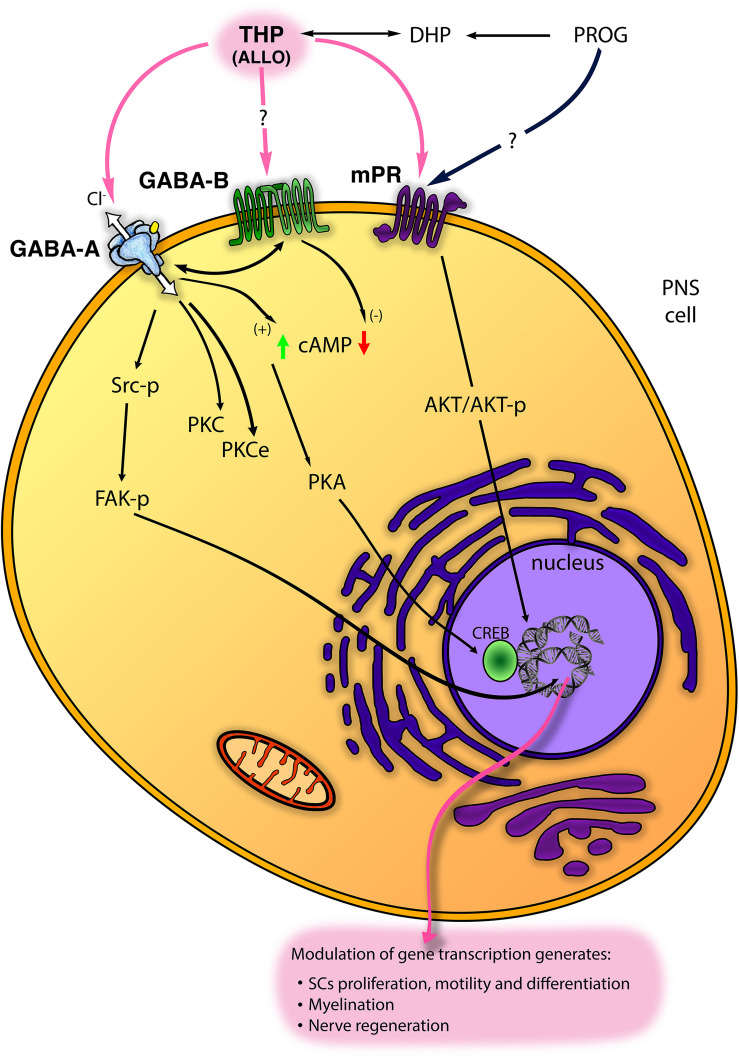
Scheme of main non-classical mechanisms of progestogens (PROG, progesterone and THP, tetrahydroprogesterone also called ALLO, allopregnanolone) in the PNS. ALLO may interact with GABA type A receptor (GABA-A), although its modulation of GABA type B receptor (GABA-B) receptor has been proposed. In SC, ALLO and likely PROG interact with the membrane PROG receptor (mPR). Some intracellular signalings downstream these receptors (including Src/FAK kinases; protein kinase C, PKC, and PKCϵ; cyclic AMP, cAMP, and protein kinase A, PKA, etc.) are reported (see text for details).

In the last decade, five subtypes of mPRs (mPRα−ε) were classified. These receptors are GPCRs, belong to the progestin and adipoQ receptor family (PAQR) and mediate rapid neuroprotective actions of progestogens (i.e., PROG and ALLO) in the nervous system (Thomas and Pang, 2012). The PR membrane component-1 (PGRMC-1; formerly named 25Dx) is another protein complexing with the plasminogen activator inhibitor 1 RNA binding protein and able to bind PROG (Peluso et al., 2008; Cooke et al., 2013). PGRMC1 was implicated in the neuroprotective effects of PROG following traumatic brain injury (Meffre et al., 2013) and spinal cord injury (De Nicola et al., 2009). Although it was found in S42 SC line (Castelnovo et al., 2019), the possible function in PNS was not further investigated. Very recently, some mPRs (primarily mPRα and mPRβ) were found in PNS and in SC *in vitro* (Figure 2), whereby they promote cell migration, proliferation and differentiation (Castelnovo et al., 2019, 2020). Indeed, in SC, mPR activation with the specific ligand O2 induced rapid downregulation of myelinating (i.e., Sox10 and Krox20) and non-myelinating [i.e., glial fibrillary acidic protein (GFAP) and neurotrophin receptor p75 (p75-NTR)] markers of SC. Contemporarily, other specific markers of repairing SC [i.e., oligodendrocyte transcription factor 1 (Olig1) and sonic hedgehog (Shh)] resulted up- and/or down-regulated following mPR activation (Castelnovo et al., 2020). These effects were mediated by an intracellular activation of phosphorylated AKT (Figure 2). Overall, these observations proved a direct control of SC by mPR, playing a promising role in the promotion of nerve re-growth (Castelnovo et al., 2019, 2020).

Recent work shed light on a peripheral glial population named “motor exit point” (MEP) glia, possessing some typical features of peripheral SC, that might be relevant in spinal cord regeneration (Fontenas and Kucenas, 2018). Theoretically, it could be speculated that mPR exerts a direct neuroprotective effect within the spinal cord through the mPR stimulation of MEP glial cells, although this hypothesis deserves further proof.

Apparently less investigated, also the mGR subfamily was studied in the PNS. Evidence of a putative non-genomic pathway including GR binding sites has been found in membrane fractions of DRG neurons, suggesting a potential rapid, GPCR-linked, non-genomic mechanism for mGR in mediating peripheral pain (Shaqura et al., 2016).

## Non-Genomic Action of Neuroactive Steroids in PNS: Involvement of Gaba and Other Receptors

Besides the steroid membrane receptors, the non-classical action of neuroactive steroids comprises the modulation of other membrane receptors, for instance, the neurotransmitter receptors GABA-A, GABA type B (GABA-B), NMDA, 5-hydroxytryptamine type 3 (5-HT_3_) and σ1 receptors (Lambert et al., 1996, 2009; Rupprecht et al., 2001; Monnet and Maurice, 2006; Sedlacek et al., 2008).

GABA-A receptor is a member of the ligand-gated ion channel family, permeable to a Cl^–^ flux and composed of five subunits from a repertoire of nineteen isoforms (i.e., α1-6, β1-3, γ1-3, δ, ε, π, θ, ρ1-3) (Whiting et al., 1995, 1997; Lambert et al., 2003). GABA-A receptor is allosterically activated by neuroactive steroids, mainly ALLO but also 3α-diol, 3β-diol and the corticosteroid 5α-3α metabolite tetrahydrodesoxycorticosterone (THDOC) (Reddy and Rogawski, 2002). In this regard, it is the most studied non-genomic mechanism of neuroactive steroids in the nervous system as well as in the PNS (Park-Chung et al., 1999; Belelli and Lambert, 2005). GABA-A receptor is classified in different subtypes based on its subunit composition. The receptor formed by one α (α2-5) plus β3 and γ2 subunits gives consistent potentiation to the ALLO-mediated GABA-activated currents (Hosie et al., 2009), while δ-containing subtype was classically described at extrasynaptic sites whereby it is highly sensitive to the 5α-3α-reduced metabolites (Mihalek et al., 1999; Belelli et al., 2002). In the PNS, GABA-A receptor is widely distributed in nerves, neurons, and glial cells. SC express the α2 and 3, β1, 2, 3 and γ2 subunits (Melcangi et al., 1999a; Magnaghi et al., 2006a), as well as the α4 and δ subunits, more characteristic of the extrasynaptic receptor (Faroni et al., 2019). In addition, we found most of these subunits in the mouse DRG neuronal cultures, with predominant expression of the synaptic subunits α1, α2, β1, β3, γ2 (Faroni et al., 2019). In general, in the PNS, the role of ALLO through GABA-A receptor has been widely characterized in the glial compartment, showing enhancement of SC proliferation, motility, differentiation and myelination (Figure 2; see also the following chapter). ALLO non-classical non-genomic effects were mimicked also by PROG and DHP, but only in prolonged (e.g., 24 h) treatment condition, when progestogens can be converted into their 5α-3α metabolite ALLO, then exerting its action via GABA-A receptor modulation (Figure 2). Recently, in DRG sensory neuron, GABA-A receptor has been characterized for its relevance in controlling peripheral pain (Chen et al., 2014; Zhang et al., 2015; Du et al., 2017), albeit ALLO’s allosteric modulation of GABA-A receptor in pain was not yet fully investigated.

Interestingly, some evidence suggests that neuroactive steroids require a different route of access to the transmembrane-domain binding sites within GABA-A receptor (Shu et al., 2004; Akk et al., 2005; Hosie et al., 2006; Chisari et al., 2009). Neuroactive steroids may be entrapped in the intracellular compartment, then re-supply the cell membrane with ligands able to modulate the GABA-A receptor at later times; by this way, the kinetic of neuroactive steroid action at GABA-A receptor may be modulated by neuroactive steroids themselves (Akk et al., 2005). In accordance, the regulation of the lipid components of the peripheral myelin may be considered as a kind of non-classical non-genomic action of neuroactive steroids in the PNS. Although this uncommon mechanism was exerted by DHP, usually acting through the classic genomic PR action, it lies in the middle ground of a genomic/non-genomic mechanism. However, in a model of diabetic neuropathy, DHP proved able to promote fatty acid desaturation and to reduce the morphological alteration of nerves, reaffirming its neuroprotective role in PNS (Mitro et al., 2014).

In principle, the possibility of ALLO to interact with the metabotropic GABA-B receptor should not be completely excluded (Figure 2). To date, although a direct interaction of ALLO with GABA-B receptor was not clearly stated, several pieces of evidence highlighted a cross-regulation between GABA-A and GABA-B receptors in PNS (Magnaghi, 2007; Faroni et al., 2019). For instance, ALLO exerted a GABA-A mediated biphasic control of different GABA-B receptor subunits (Magnaghi et al., 2006a; Magnaghi, 2007). The metabotropic GABA-B receptor is a dimeric complex, a member of the GPCR superfamily (Bowery and Enna, 2000). In the PNS, GABA-B receptor subunits 1a, 1b and 2 were found in SC, sciatic nerve, satellite cells and DRG neurons (Magnaghi et al., 2004; Magnaghi, 2007; Faroni et al., 2014), where the functional receptor was proved to be negatively coupled to the adenylate cyclase system (Figure 2; Magnaghi et al., 2004). Its activation decreased SC proliferation and the expression of some important myelin proteins, like P0, PMP22, MAG and connexin 32 (Magnaghi et al., 2004). In a neuropathic model of partial sciatic ligation, a 7-day administration of specific GABA-B ligands (i.e., baclofen and the antagonist CGP56433) strongly improved the biochemical, morphological and behavioral outcomes of sciatic nerve (Magnaghi et al., 2014). Furthermore, studies in transgenic mice with a conditional deletion of GABA-B1 receptor in PNS demonstrated that some important GABA-A subunits, expressed in SC and DRG neurons, were cross-regulated by GABA-B receptor (Faroni et al., 2019).

Beside the GABA-A receptor, other neurotransmitter receptors, such as NMDA receptor, are affected by neuroactive steroids (Figure 1). Indeed, it was shown that PREG, DHEA and DHEA sulfate activate allosterically NMDA receptor (Figure 1), while PREG sulfate acts as the negative modulator (Wu et al., 1991; Baulieu, 1997; Park-Chung et al., 1999). NMDA is an ionotropic glutamate receptor also distributed in the PNS, where it localizes in peripheral axons and SC (Evans et al., 1991, 1992; Carlton et al., 1998; Christensen et al., 2016; Campana et al., 2017). The PNS also has specific glutamate transporters and it synthesizes glutamate, which was found in sensory and motor neurons (Chen et al., 2017). Moreover, sensory cranial ganglia synthesize glutamate (Malet and Brumovsky, 2015), while the SC possess the enzymatic machinery able to uptake and synthesize glutamate, like the excitatory amino acid transporter 1 (EAAC1) and glutamine synthetase (Miller et al., 2002; Perego et al., 2012). The σ1 receptor is an intracellular protein that localizes in membranes of the endoplasmic reticulum, plasmalemma, nucleus, and mitochondria (Alonso et al., 2000) and it is able to enhance NMDA activity (Pabba and Sibille, 2015). Activation of this receptor rises intracellular Ca^++^ influx via NMDA (Hayashi et al., 2000), thus confirming the capability of σ1 to modulate NMDA receptor. Accordingly, it was shown that DHEA sulfate acts as σ1 agonist, inducing a clear σ1-like potentiation of NMDA response, while PREG sulfate exerted opposite effects; also PROG is an endogenous antagonist of σ1 receptor (Maurice et al., 1999). To date, evidence of neuroactive steroid actions through NMDA or σ1 receptor, specifically in the PNS, has not been yet provided.

## Allo Activation of Gaba-A Receptor in PNS: Intracellular Signaling

ALLO is the most re-known neuroactive steroid able to regulate the PNS, controlling glial proliferation, differentiation and myelination processes (Figure 2). ALLO non-genomic effects on GABA-A receptors usually occurred at nanomolar concentration, engaging an allosteric interaction that entails the presence of the endogenous ligand GABA. Instead, at high concentration (micromolar range) ALLO directly gates GABA-A receptor (Callachan et al., 1987), although it was shown that neuroactive steroids might directly gate GABA-A receptor even at 100 nM, likely attaining a relatively low kinetic (Shu et al., 2004).

In any case, the presence of endogenous GABA is a requisite for ALLO action. Following the former observation in the early 1980’s proving GABAergic fibers in PNS (Brown and Marsh, 1978; Morris et al., 1983; Olsen et al., 1984), it was unequivocally demonstrated the presence of GABA and its synthetic machinery (glutamic acid decarboxylase of 67 kDa, GAD67) in SC (Magnaghi et al., 2010). An autocrine loop has been proposed, through which nanomolar concentration of ALLO was able to increase the GAD67 levels in SC, thus providing GABA as the endogenous ligand for the GABA-A receptor (Magnaghi et al., 2010). Therefore, the local GABA synthesis in peripheral nerves supports the allosteric action of ALLO in SC and neighboring compartments. In accordance, it was shown that SC possesses EAAC1, the active uptake system able to provide glutamate as a precursor for GABA synthesis (Perego et al., 2011, 2012). In SC, EAAC1 expression and activity were still controlled by ALLO, through a GABA-A mediated and protein kinase C (PKC) mechanisms (Figure 2) ALLO promoted the transport of EAAC1 from the intracellular stores into the SC membrane (in actin-rich cell tips), modifying their morphology (Perego et al., 2012).

ALLO was shown to increase SC proliferation (Perego et al., 2012; Melfi et al., 2017) and this action was GABA-A mediated because it was mimicked by the specific ligand muscimol and blocked by the specific antagonist bicuculline (Perego et al., 2012). It was highlighted that ALLO’s control of SC proliferation was dependent on EAAC1 transport and activity at SC plasma membrane (Perego et al., 2012), once again confirming that GABA synthesis was necessary for ALLO effects. ALLO also stimulated morphologic changes and motility of SC, then promoting myelination (Melfi et al., 2017), which are fundamental processes for the development, maturation, and regeneration of PNS. Remarkably, ALLO participated in the control of peripheral myelin proteins (e.g., P0, MAG), being particularly active in enhancing the levels of PMP22, mRNA and protein (Melcangi et al., 1999a, b; Magnaghi et al., 2001). The specificity of this action was confirmed, respectively, by the capability of muscimol to replicate and of bicuculline to abolish the ALLO’s effects on PMP22 (Magnaghi et al., 2001, 2006a). This confirmed the hypothesis that in SC protein PMP22 is controlled by GABA-A receptors. However, the capability to stimulate PMP22 expression was observed also with 3α-diol (Magnaghi et al., 2001), likely via the same allosteric GABA-A receptor modulation (Frye et al., 1996; Figure 1).

As expected, the intracellular mechanisms downstream the ALLO modulation of GABA-A receptors imply changes in intracellular Cl^–^ flux (Figure 2). Conversely, at least in PNS, most of ALLO effects reflected as transcriptional changes. In the last decades, some studies were addressed to clear this point. For instance, in the developing rat cortex, GABA-A receptor activation leads to an increase of Ca^++^ influx through L-type voltage-gated Ca^++^ channels. This leads to the phosphorylation and activation of the cAMP response element-binding protein (CREB) transcription factor, in turn regulating protein expression, for instance of the brain derived neurotrophic factor (Mantelas et al., 2003). Unfortunately, these mechanisms were not shown in the PNS, whereas the concomitant activation of ion channels (e.g., Ca^++^ channels), following neuroactive steroid binding to GABA-A receptor, is still questionable. Although the involvement of Ca^++^ channel is not clear, ALLO was proved able to modulate the protein kinase A (PK-A), through enhanced cAMP levels and CREB phosphorylation (Magnaghi et al., 2010), or the PKC pathway (Perego et al., 2012; Figure 2). These intracellular signalings were supposed to be downstream the allosteric action of ALLO at GABA-A receptor. Recently, another intracellular ALLO’s pathway has been found. ALLO effects on SC proliferation, motility and myelination, indeed, imply tyrosine protein kinase Src (Src) and focal adhesion kinase (FAK) activation (Melfi et al., 2017), although other signaling pathways should not be excluded (Figure 2). ALLO effects on Src were mimicked by muscimol, counteracted by bicuculline and by the specific Src inhibitor PP2, suggesting that in SC ALLO activation of GABA-A induces an intracellular phosphorylation cascade, leading to actin rearrangements of the cytoskeleton, enhancement of SC motility and myelination (Melfi et al., 2017; Figure 2).

One strategy that neuroactive steroids use to control GABA-A receptor function is to phosphorylate/de-phosphorylate its subunits by the recruitment of protein kinases or phosphatases (Belelli and Lambert, 2005). Phosphorylation of GABA-A can produce different effects, ranging from enhancement to inhibition, depending on the subunit targeted and on the location of sites being phosphorylated (Moss and Smart, 1996). In parallel, PKC phosphorylation of GABA-A receptor may influence the sensitivity to neuroactive steroids (Brussaard and Koksma, 2003; Vergnano et al., 2007). Accordingly, PKC-ε is considered as a novel isoform of PKC, regulating the sensitivity to neuroactive steroids. Indeed, animals lacking PKC-ε showed hypersensitivity to behavioral effects induced with allosteric GABA-A receptor modulation (Hodge et al., 2002). PKC-ε was found in SC and DRG neurons in culture (Puia et al., 2015). Interestingly, PKC-ε was upregulated in DRG neurons exposed to the culture medium from ALLO-treated SC, suggesting that these cells release one or more factors able to regulate PKC-ε in DRG neurons (Puia et al., 2015). Since PKC-ε is relevant in modulating some pain pathways, we speculated that these mechanisms identified novel putative circuits involved in the control of pain processes at PNS and spinal cord levels (Puia et al., 2015).

In the PNS, ALLO hired importance also during neurodegenerative conditions, likely implying the regulation of other nervous cells or structures (i.e., DRG neurons). In a model of STZ-induced diabetic neuropathy, ALLO and 3α-diol enhanced nerve conduction velocity (NCV) and intraepidermal nerve density, decreasing sensitivity to thermal pain (Leonelli et al., 2007; Roglio et al., 2007). Although the mechanism behind these effects was not elucidated, the involvement of non-genomic mechanisms through GABA-A receptor was hypothesized. In support of the non-genomic action of ALLO in neuropathic pain (Patte-Mensah et al., 2014), it should be highlighted that ALLO may regulate other channels and/or signaling pathways involved in neuropathic pain, such as T-type Ca^++^ channels, voltage-gated Na^+^ channels, purinergic receptor P2X3 and bradykinin signaling (Cho and Chaban, 2012; Ayoola et al., 2014).

## Conclusion

In this review, we sum up most of the latest evidence on the effects of neuroactive steroids, either classical or non-classical, genomic or non-genomic, in the PNS. Neuroactive steroids exhibit important functions in the development, myelination, neuroprotection and nerve repair of the PNS. In particular, ALLO revealed the most well studied and incisive neuroactive steroid in regulating the biologic and physiologic functions of the PNS. Here we reported several steps forward in the identification of its mechanism of action. Some ALLO’s effects may be ascribed to GABA-A (or likely GABA-B) activation, PKA, PKC or PKC-ε modulation, as well as to the Src/FAK kinases involvement. Besides, mPR or electrophysiological changes in ion channels, likely Cl^–^ flux, have been recently proposed to occur also in the PNS.

Interestingly, the neuroregenerative effects of ALLO via GABA-A receptor might be promising for the treatment of the peripheral neurodegenerative pathologies, particularly for traumatic injuries requiring the surgical application of bioengineered conduits. Indeed, *in vitro* testing of 2D silk fibroin scaffold, functionalized for controllable *in situ* delivery of ALLO, showed great potential for nerve repair (Gennari et al., 2018). Therefore, the administration of neuroactive steroids might represent a novel and promising strategy to prevent or treat different types of peripheral neuropathies and the associated neuropathic pain.

## Author Contributions

AC wrote and revised the whole manuscript. VB prepared the figures and revised the manuscript. VMe searched the bibliography and proofread the manuscript. VMa planned, wrote and revised the whole manuscript. All authors contributed to the article and approved the submitted version.

## Conflict of Interest

The authors declare that the research was conducted in the absence of any commercial or financial relationships that could be construed as a potential conflict of interest.

## Abbreviations

3 α-diol: 5 α-androstane -3 α,17 β-diol
3 β-diol: 5 α-androstane -3 β,17 β-diol
3 α-HSD: 3 α-hydroxysteroid dehydrogenase
3 β-HSD: 3 β-hydroxysteroid dehydrogenase
5 α-R: 5 α-reductase
5HT_3_: 5-hydroxytryptamine type 3
ALLO: allopregnanolone
AR: androgen receptor
CREB: cAMP response element-binding protein
DHEA: dehydroepiandrosterone
DHP: dihydroprogesterone
DHT: dihydrotestosterone
DRG: dorsal root ganglia
E2: 17 β-estradiol
EAAC1: excitatory amino acid transporter 1
EGR1/Krox-24: early growth response (EGR1/Krox-24)
EGR2/Krox-20: early growth response 2 (EGR2/Krox-20)
Egr3: early growth response 3
ER: estrogen receptor
ERK1/2: extracellular signal-regulated protein kinase 1 and 2
FAK: focal adhesion kinase
GABA: γ-aminobutirric acid
GABA-A: GABA type A receptor
GABA-B: GABA type B receptor
GAD67: glutamic acid decarboxylase of 67 kDa
GFAP: glial fibrillary acidic protein
GPCR: G protein-coupled receptor
GPR30: GPCR ER-1 or GPER1
GR: glucocorticoid receptor
LXR: liver X receptor
MAG: myelin associated glycoprotein
mAR: membrane androgen receptor
MEP: motor exit point
mER: membrane estrogen receptor
mGR: membrane glucocorticoids receptor
mPR: membrane progesterone receptor
MR: mineralocorticoid receptor
NCV: nerve conduction velocity
NMDA: N-Methyl -D-aspartate
Olig1: oligodendrocyte transcription factor 1
P0: glycoprotein P0
P450C17: 17 α -hydroxylase/17,20-lyase
P450scc: P450 side-chain cleavage enzyme
p75-NTR: neurotrophin receptor p75
PAQR: progestin and adipoQ receptor family
PGRMC-1: PR membrane component-1
PHTPP: 2-Phenyl-3-(4-hydroxyphenyl)-5,7-bis(trifluoromethyl)-pyrazolo[1,5-a]pyrimidine
PK-A: protein kinase A
PK-C: protein kinase C
PMP22: peripheral myelin protein 22
PNS: peripheral nervous system
PR: progesterone receptor PREG pregnenolone
PROG: progesterone
SC: Schwann cells
Shh: sonic hedgehog
Src: tyrosine protein kinase Src
SRC1: steroid nuclear receptor coactivator
Sox-10: SRY-box10
StAR: steroidogenic acute regulatory protein
STZ: streptozotocin
T: testosterone
THDOC: tetrahydrodesoxycorticosterone
THP: tetrahydroprogesterone
TSPO: translocator protein.
